# Retraction: Zoledronic acid generates a spatiotemporal effect to attenuate osteoarthritis by inhibiting potential Wnt5a-associated abnormal subchondral bone resorption

**DOI:** 10.1371/journal.pone.0340043

**Published:** 2026-01-08

**Authors:** The PLOS One Editors

After this article [1] was published, concerns were raised about Figs 2 and 6. Specifically, the following figure panels appear similar:

- Fig 2A 8 weeks DMM+PBS panel of [1] and Fig 8B 8 weeks Sham-operated coronal panel of [2].
- Fig 6A sham+PBS panel of [1] and Fig 3D day 3 RANKL-100 ng/ml CUR-10 μM panel of [3, retracted in 4].

Upon editorial follow-up, data underlying the panels of concerns were provided; however, these data were not sufficient to resolve the concerns listed above and raised further concerns about the reliability of the data underlying the results presented in this article.

PLOS additionally noted concerns about adherence to PLOS policy on Animal Research. Specifically, the ethical approval document submitted with [1] is dated after the study period during which the animal research was conducted. The first author indicated that the study protocol received ethical approval at an earlier date, but the paper version of the ethical approval document was issued after the work was completed. The *PLOS One* Editors consider this issue unresolved in the absence of documentation confirming ethical approval of the work in advance of the study start date and we regret that these issues were not addressed prior to the article’s publication.

In light of the above unresolved concerns, the *PLOS One* Editors retract this article.

All authors agreed with the retraction and apologize for the issues with the published article.The Fig 2A 8 weeks DMM + PBS panel appears similar to content previously published in [2] under a CC BY 4.0 license by Spandidos Publications. The Fig 2A 8 weeks DMM + PBS panel is subject to the license that applies to the original article.
